# Breast Imaging of Transgender Individuals: A Review

**DOI:** 10.1007/s40134-018-0260-1

**Published:** 2018-01-18

**Authors:** Emily B. Sonnenblick, Ami D. Shah, Zil Goldstein, Tamar Reisman

**Affiliations:** 1 Department of Radiology, Icahn School of Medicine at Mt Sinai, One Gustav Levy Place, New York, NY 10029 USA; 2 Department of Medicine, Center for Transgender Medicine and Surgery at Mt. Sinai , 275 Seventh Avenue, New York, NY 10011 USA; 3 Department of Medicine, Icahn School of Medicine at Mt. Sinai, 275 Seventh Avenue, New York, NY 10011 USA

**Keywords:** Transgender, Breast, Mammography, Transsexual, Screening

## Abstract

**Purpose:**

This review will inform radiologists about the evidence base regarding radiographic imaging for transgender individuals and considerations for providing culturally sensitive care for this population.

**Findings:**

Transgender individuals are increasingly referred for both screening and diagnostic breast imaging. It is important that the clinic environment is welcoming, the medical staff utilize accepted terminology and patients are able to designate their gender and personal history to ensure appropriate care. Hormone and surgical treatments used for transition by many transgender women and men may change the approach to imaging.

**Summary:**

Although not yet evidence-based, screening mammography is currently suggested for transgender women with risk factors, including those receiving hormone treatment over 5 years. The risk for breast cancer in transgender individuals is still being defined.

## Introduction

It is estimated that from 8 to 25 million individuals worldwide now identify as transgender [1••]. The increasing use of cross hormone treatment and sex reassignment surgery to affirm gender identity in this population poses special considerations for the radiologist. Some clinical scenarios translate seamlessly from the cisgender population to the transgender patient. For instance, when a transgender patient is referred for diagnostic imaging for a breast complaint such as a palpable mass, the breast imager will generally employ the same protocols used for non-transgender individuals. However, other scenarios are more complicated. In particular, there is a lack of clarity with regards to indications for breast cancer screening in transgender women.

In this paper, we will present indications for diagnostic breast imaging in the transgender population, show expected radiographic findings, and provide an evidence-based review of the current recommendations for breast cancer screening in transgender individuals based on relative risks for cancer derived from both retrospective and cohort studies. We will also outline practical changes that should be considered to provide culturally sensitive health care to this population. A conclusion that will emerge from this review is that there remains a need for prospective longitudinal follow-up of transgender individuals electing breast cancer surveillance; a description of and a means to refer patients to one such registry will be presented.

## Definitions/Terminology

Transgender is an adjective used to describe an individual whose gender does not correspond to the one assigned to them at birth. It is independent of genotype, sexual orientation, and behavior. While gender assigned at birth is assessed by inspection of genitalia, gender identity arises from an individual’s internal sense of being male or female or something else. Individuals assigned male sex at birth who desire to live as female (transgender women, male to female, MTF) or assigned female sex at birth who desire to live as male (transgender men, female to male, FTM) may take steps to alter their outward appearance to align with their gender identity. Transition refers to the process of changing gender expression or physical appearance to align with gender identity. Gender reassignment refers to steps taken to permanently alter one’s phenotype and can be achieved by hormone treatment and surgery. Cisgender and non-transgender refer to people whose gender and gender expression aligns with that assigned to them at birth [2]. Table 1 gives a breakdown of each term and definition.

**Table 1:** Definitions/Terminology

Term	Definition
Gender identity	Arises from an individual’s internal sense of being male or female or something else
Transgender	Adjective to describe an individual whose gender does not align with that assigned to them at birth
Transgender woman	A person who was assigned male sex at birth and identifies as female
Transgender man	A person who was assigned female sex at birth and identifies as male
Transition	Refers to steps taken to alter outward appearance to align with gender identity
Gender reassignment	Refers to steps taken to permanently alter one’s phenotype and can be achieved by hormone treatment and surgery
Cisgender or non-transgender	Adjectives to describe a person whose gender and gender expression aligns with that assigned to them at birth

## Cultural Sensitivity

Due to stigma and discrimination, transgender individuals have suffered from lack of access to competent medical care [3, 4]. Given the growing desire among health-care professionals to improve access for the transgender community, it is critical for those in the medical community to develop methods for delivering culturally sensitive care in a welcoming environment [5]. Intake forms should allow the patient to self-identify gender, their preferred name, pronoun, and information about gender affirming medical or surgical treatment. This will allow for more complete and accurate information to be entered into standard data fields in medical records thus facilitating delivery of culturally sensitive care and allowing providers to address health care concerns specific to this population of patients which might otherwise be overlooked. Centers that embrace diversity should avoid gender-specific signage (e.g., Women’s Imaging Center). Bathroom facilities should be gender neutral. Privacy may be ensured by providing private changing rooms or allowing the patient to change in the exam room.

## Medical Hormone Treatment

### Transgender Women

An essential element of transition for transgender women is breast development. Transgender women may be treated with estrogen for feminization. In addition to estrogen, agents with antiandrogen activity such as spironolactone may be used to suppress testosterone [6, 7•].

**Fig. 1:**
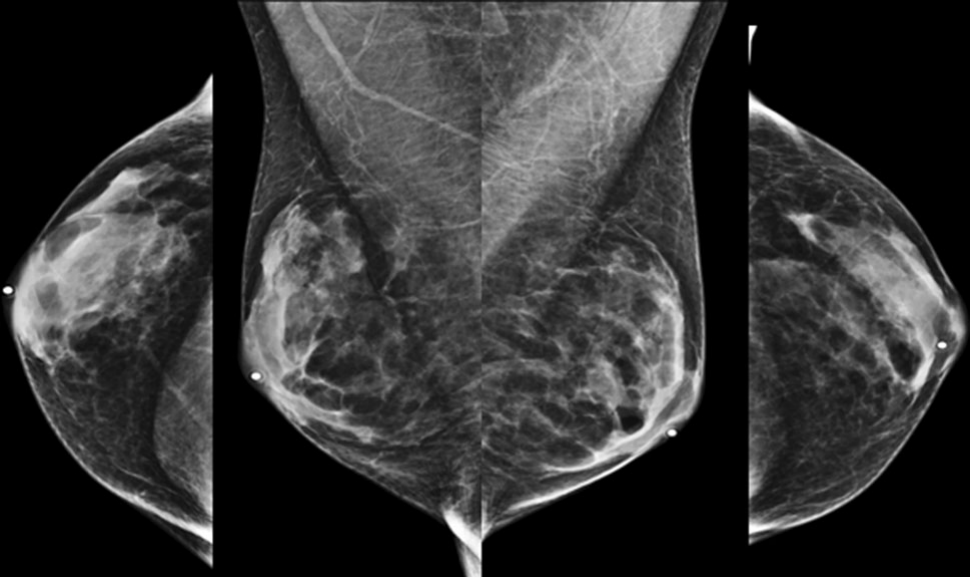
A 61-year-old transgender woman treated with estradiol and spironolactone for over 10 years. Standard CC and MLO views from her screening mammogram show normal heterogeneously dense breasts

Breast development secondary to a physiologic estrogen surge in natal girls has been categorized into five Tanner stages of pubertal development [8]. Transgender women experience muted Tanner stages. There is an initial development of a subareolar breast bud at 3–6 months followed by further enlargement and development of the breast. Maximal breast growth is realized at 2–3 years in our experience. Figure 1 shows heterogeneous breast tissue in a mammogram from a transgender woman treated with cross-sex hormones. Breast size and tissue composition following estrogen treatment varies for each individual. [9•] Transgender women are unlikely to reach Tanner stage 5 [6]. The degree of breast development seems to be independent of type and dose of hormone treatment [9•]. Adding progestins to estrogen does not appear to alter breast size [9•]. Approximately 60% of trans women seek breast augmentation surgery regardless of the type of estrogen used for feminization [9•, 10]. Augmentation performed by established surgical standards in the U.S. employs silicone or saline implant in pre-pectoral or retro-pectoral location. While illegal in the U.S., some individuals may have had free injections of a number of substances such as free liquid silicone for purposes of augmentation.

### Transgender Men

Transgender men may be treated with testosterone for masculinization and elect surgery to create a male chest contour.

Testosterone is delivered by intramuscular injection or by transdermal patch, gel, or implant [6, 7•]. Progestins may be added to curtail menses and prevent endometrial hyperplasia. Transgender males taking testosterone will demonstrate serum testosterone levels in the mid to normal male range. In our personal experience, serum estradiol levels may be maintained in post-menopausal female reference range. Some studies have shown estradiol levels above normal male levels in 71% of transgender men at 6 months of treatment [11]. The potential for elevated estrogen has been hypothesized to reflect peripheral aromatization of circulating testosterone [12].

Transgender men who elect surgical breast removal, referred to as “top surgery”, may have residual breast tissue if the surgical procedure consists of contouring of the chest and nipple sparing. It is generally agreed that transgender male patients who undergo breast removal are at very low risk of developing breast cancer. Models for breast cancer risk reduction following simple mastectomy are derived from high-risk cisgender women. It has been observed that there is less than 2% risk for breast cancer in cisgender women at increased hereditary risk who have prophylactic mastectomy [13].

## Histologic Changes from Hormone Treatment

### Transgender Women

**Fig. 2:**
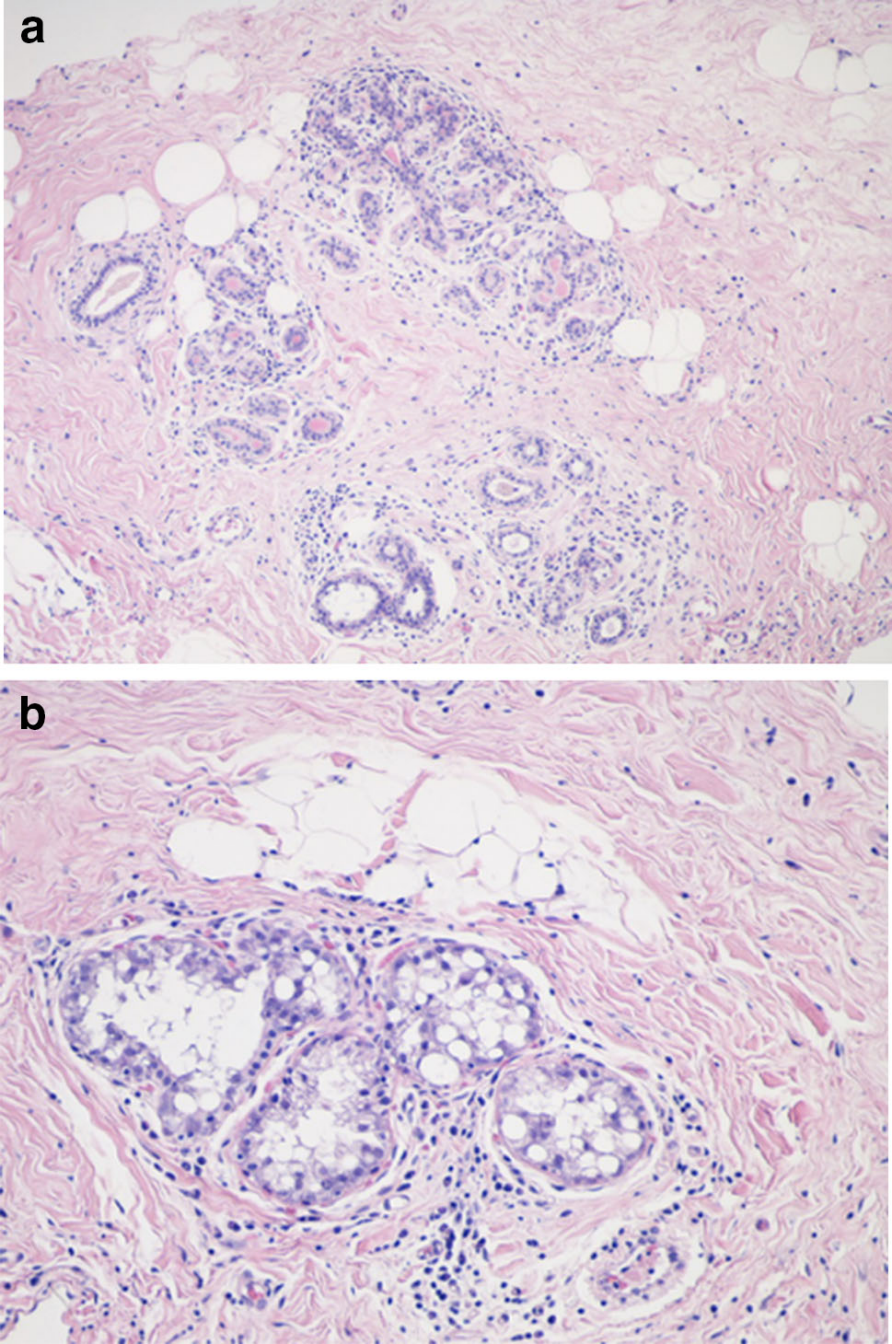
Histology from the breast of a 55-year-old transgender woman treated with cross-sex hormones since age 19. **a** Lobule formation (H and E stain at ×100 magnification). **b** Pseudolactational changes (H and E stain at ×200 magnification)

A mildly elevated ratio of estrogen to progesterone may normally occur in cisgender males during infancy, adolescence and advanced age [14]. This causes variable degrees of proliferation of ductal epithelium which is visualized radiographically as dense tissue centered behind and extending from the nipple referred to as gynecomastia [15]. Males with prostate cancer treated with androgen deprivation develop heterogeneously dense breast tissue referred to as diffuse gynecomastia. This correlates heterogeneously dense tissue correlates histologically with moderate acinar and lobular development [16]. The histologic effect of high levels of estrogen utilized for transition from male to female, unlike gynecomastia, includes development of ducts, lobules and acini histologically identical to cisgender women. Pseudolactational changes have also been described [16]. Figure 2 shows an example of lobule formation and pseudolactational changes in the breast biopsy from a transgender woman. We have also observed lobular development similar to a pre-pubertal breast in the setting of estrogen treatment of transgender females [17].

### Transgender Men

Histologic changes due to progesterone or androgen given in the setting of female to male transition have been inconsistent between studies. Slagter’s group looked at histology from 23 trans men treated with injectable testosterone and observed reduced glandular tissue and increased fibrous connective tissue similar to involutional changes observed in post-menopausal women [18]. However, fibrocystic lesions such as cysts, adenosis and duct and lobular hyperplasia found in post-menopausal women were rarely observed in transgender male breast tissue [18]. There are also immunohistochemical differences in the breast tissue of transgender men [19]. Increased fibrous stroma and lobular atrophy have been observed in transgender men receiving long-term testosterone [19]. In another study, only microcalcifications and no other significant changes in breast histology and immunochemistry were noted in mastectomy specimens from 29 transgender men on long-term androgens [20].

Finally, the largest study of mastectomy specimens from 100 transgender males—who were of average age 28 and received androgens for 2–9 years prior to surgery—showed markedly reduced glandular tissue and proliferation of fibrous stroma in 93% of cases [21]. These investigators observed fibrocystic lesions in 34 cases and 2 fibroadenomas. Of interest, there were no cases of atypical hyperplasia, in situ carcinoma or features of gynecomastia. [21]

## Imaging Appearance

### Transgender Women

Cross-sex hormone treatment for transgender women causes development of ductal epithelium and lobules which vary in distribution and density between individuals. The same breast pathology that occurs in natal women should be expected in transgender women. There are reports of benign entities such as fibroadenomas [22, 23], lipomas and angiolipoma [24] as well as malignancies including a malignant phyllodes tumor [25] imaged in transgender women treated with cross hormone therapy [26].

**Fig. 3:**
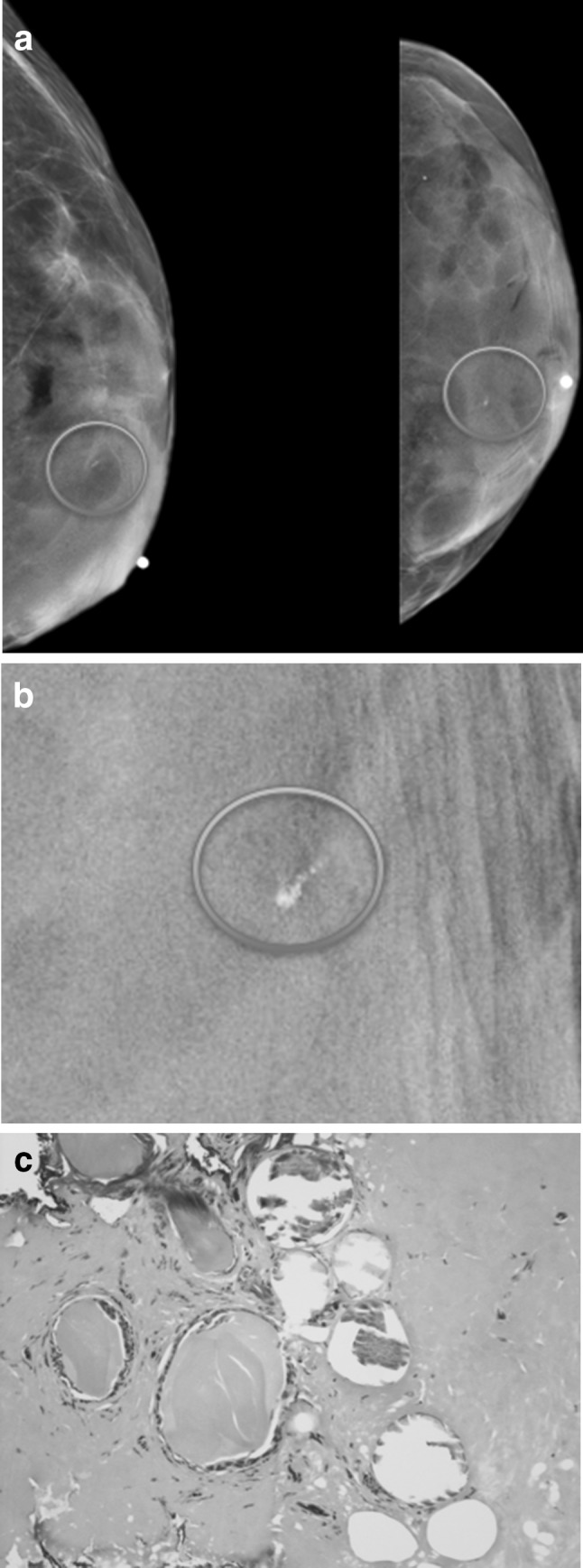
A 57-year-old transgender woman treated with estrogen for 37 years. **a** Left breast mammogram shows extremely dense breast tissue containing a group of calcifications (circled). **b** Magnified view shows pleomorphic calcifications. **c** Histopathology from stereotactic biopsy shows tangential section of epithelial lined cystic ducts in fibrous stroma (H and E stain 400X). Findings are consistent with fibrocystic changes

A Belgian series assessed 50 transgender women who were at least 6 months post-sex reassignment surgery with screening mammography and ultrasound [27]. In this series, 94% of transgender female patients were on estrogen therapy; however, the duration of hormone treatment was not recorded. Of these 50 patients, 60% were judged to have over 25% dense tissue. There was a significant correlation between degree of breast density on mammography and ultrasound. A single fibroadenoma, several cysts, and two lipomas were detected by ultrasound. Imaging features of these benign lesions were identical to those in cisgender women. Figure 3 shows a group of indeterminate calcifications found on a screening mammogram which underwent biopsy which yielded fibrocystic changes in our practice.

**Fig. 4:**
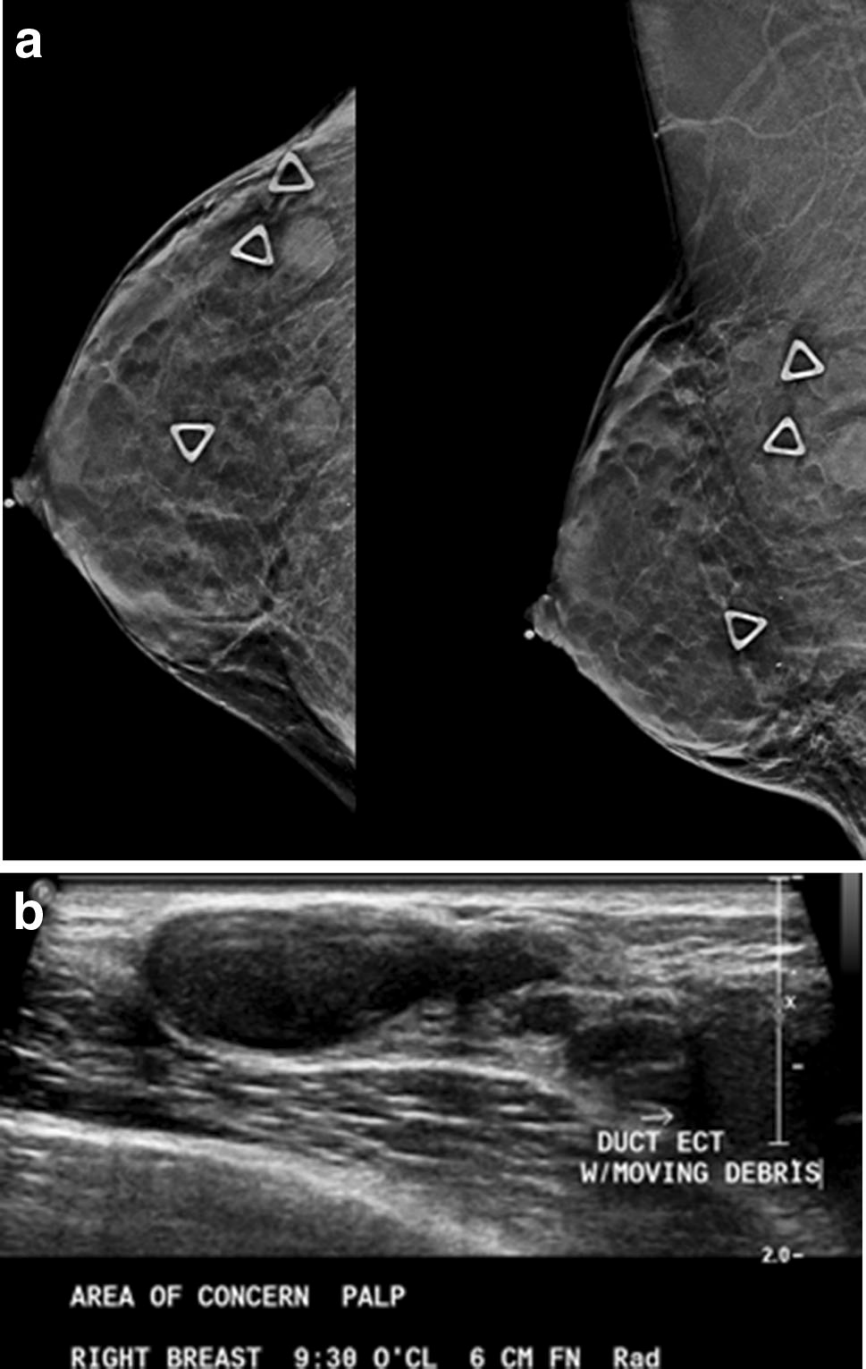
A 58-year-old transgender woman on hormone treatment complains of lumps in her breast. **a** CC and MLO mammography views show triangle-shaped skin markers over palpable masses. **b** Ultrasound shows benign duct ectasia which correlates with the palpable masses

Diagnostic imaging using mammography, ultrasound or MRI is the same in transgender and non-transgender women. Palpable abnormalities, in the absence of free injected silicone, can be evaluated with mammography and ultrasound. Figure 4 shows palpable benign duct ectasia with massive dilated ducts containing debris demonstrated on both mammogram and ultrasound. While bilateral clear nipple discharge is a physiologic finding, unilateral clear or bloody discharge may warrant imaging with mammography followed by breast ultrasound for patients over age 30. As with cisgender women, initial imaging for pathologic nipple discharge or palpable abnormality using ultrasound is suggested for those under age 30 [28].

In our experience, immature lobules similar to those found in adolescent breast tissue were noted in a transgender women at age 65 who had been taking estrogen for 13 years [17]. Therefore, it is theoretically possible that breast tissue may remain immature and sensitive to ionizing radiation in transgender women who start hormone treatment as adults. Further study is needed to validate this hypothesis and may influence future recommendations for age at which to start with breast ultrasound verses mammography in this population.

**Fig. 5:**
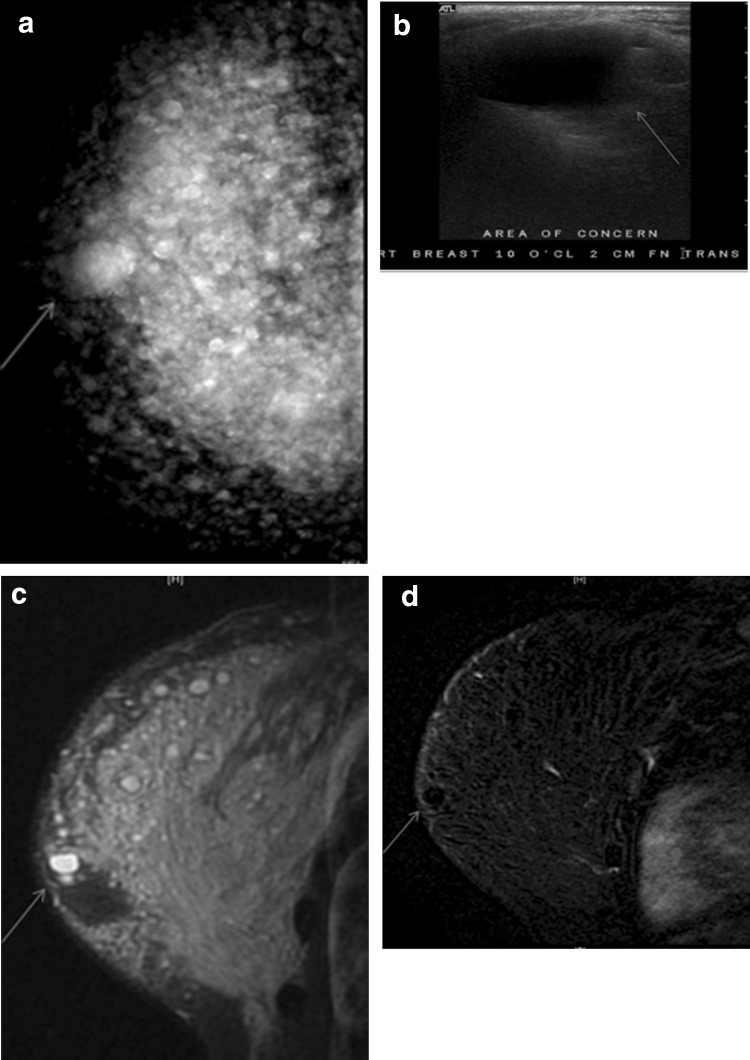
A 60-year-old transgender woman with free silicone injections feels a lump. **a** Mammogram shows multiple high-density masses which are silicone granulomas. The palpable granuloma is designated by blue arrow. **b** Ultrasound shows the palpable silicone granuloma is a circumscribed anechoic mass (arrow). **c** The silicone granuloma is a very high signal circumscribed mass on MRI T2 STIR sequence (arrow). **d** On a TI post-contrast sequence the silicone granuloma is a non-enhancing circumscribed mass (arrow)

**Fig. 6:**
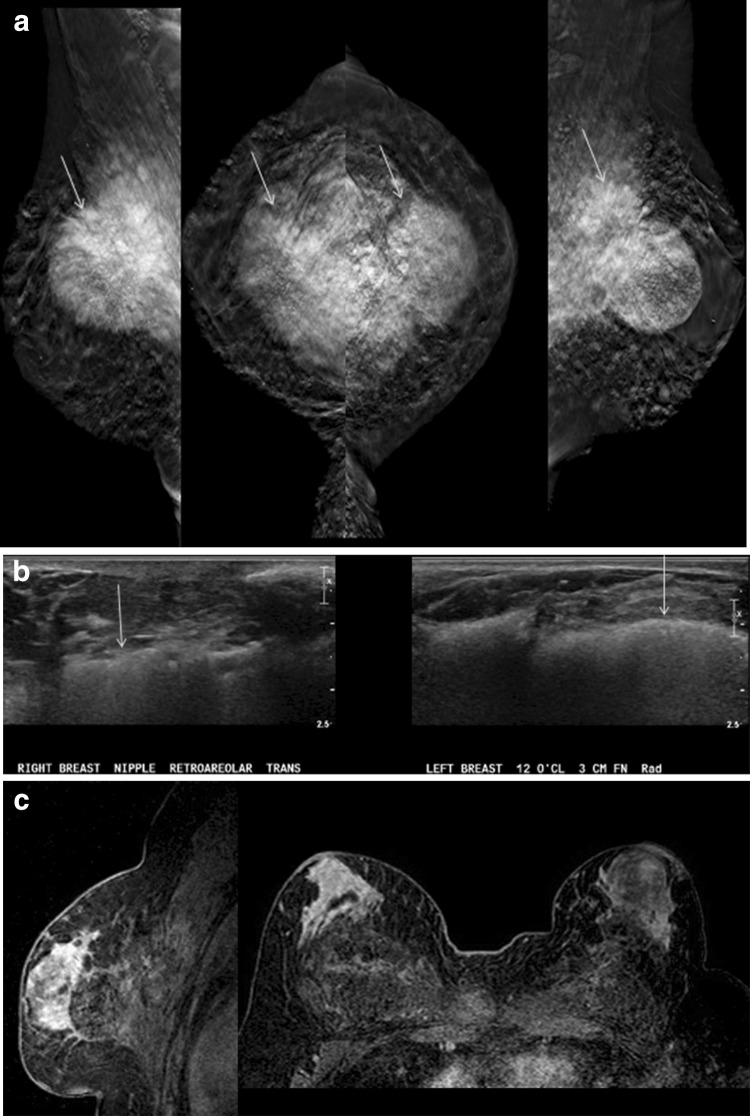
A 54-year-old transgender woman is referred for a screening mammography prior to nipple sparing mastectomy to remove painful masses caused by free silicone. **a** Tomosynthesis shows bilateral large dense masses which are inseparable from the pectoralis muscle (arrows). There are also innumerable small silicone granulomas. **b** Ultrasound shows an example of the indistinct hyperechoic “snow storm” appearance of free silicone at posterior depth in both breasts (arrows). **c** Post-contrast subtracted MRI image shows no suspicious enhancing mass

As with cisgender patients, breast implants are imaged with standard oblique and craniocaudal views and Eklund displaced views. Breast augmentation by direct injection of particles such as silicone, mineral oil, liquid paraffin, or petrolatum jelly presents a special challenge for imaging. This material migrates in the fat and muscle resulting in masses termed sclerosing lipogranulomas [29–32]. Breast lumps, inflammation, pain and physical disfiguration cause individuals to seek medical care. Fibrosis and granulomas obscure normal tissue on mammography and ultrasound. On mammography these free particle injections present as numerous diffuse round and irregular high-density masses which represent fibrotic granulomas. Figure 5 shows palpable silicone granulomata which vary in size imaged by mammography, ultrasound and MRI. Free silicone may also create large fibrotic masses in the retroglandular fat and pectoralis muscle which mimic malignancy and obscure breast tissue as displayed in Fig. 6 [33]. Contrast enhanced breast MRI is the preferred mean for detecting cancer in these patient with free particle injections. On breast MRI the granulomas are non-enhancing circumscribed T2 high signal with absent signal on T1-weighted fat-suppressed images.

**Fig. 7:**
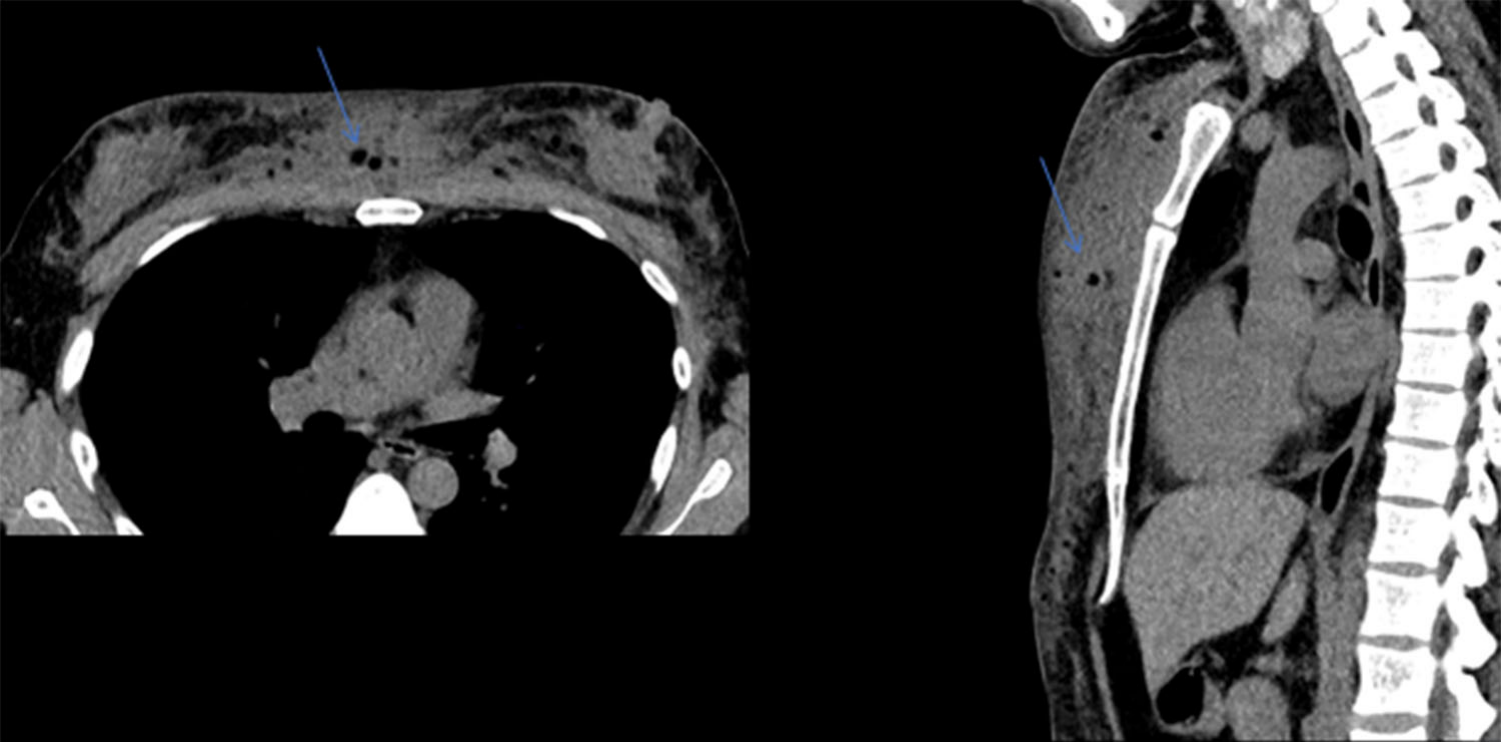
Chest wall migration of mineral oil incidentally seen on a CT scan in a 41-year-old transgender woman with a history of breast augmentation with free mineral oil self-injections (arrows)

Free oil injections may be detected on cross-sectional imaging. Diffuse circumscribed fat density masses in the chest wall in Fig. 7 are secondary to migration of mineral oil injected into the breast for augmentation purposes.

## Hormones and Breast Cancer Risk

The relationship of altered androgen and estrogen on breast cancer risk is poorly understood. The risk for breast cancer due to exogenous hormones prescribed for transgender individuals is informed by studies of estrogen and androgens in the general population. Use of relatively short-term combined exogenous estrogen and progesterone in post-menopausal cisgender women was associated with increased breast cancer incidence; however, exogenous estrogen alone was not associated with increased risk, according to data from the Women’s Health Initiative [34]. According to an analysis of nine prospective studies of endogenous hormone levels and breast cancer risk in post-menopausal women, breast cancer is associated with elevated levels of circulating estrogen and androgens in post-menopausal women [35–37]. Based on these studies, it is reasonable to hypothesize that breast cancer risk might be elevated for transgender women treated with hormone replacement therapy.

Conversely, though there are abundant androgen receptors in normal breast tissue and androgen receptors are also frequently expressed in breast cancers, there is no evidence for increased breast cancer incidence in women with hyperandrogenism such as polycystic ovary syndrome, or in transgender men receiving testosterone treatment [21]. This is somewhat counterintuitive, as there is aromatization of androgens to estrogen in the peripheral blood of transgender men on testosterone, which may result in maintenance of estradiol levels [12]. Breast cancer risk in transgender women is potentially reduced due to a relatively shorter duration of lifetime exposure to estrogen compared to cisgender women. It has been demonstrated that early menarche and late menopause are associated with increased risk of breast cancer in cisgender women [38].

### Prospective and Retrospective Cohort Analysis

It is not possible to derive cancer incidence in transgender individuals from existing U.S. cancer registries because transgender status is not documented. Misclassification of sex/gender assignment in U.S. cancer registries is a well-known phenomenon which may also result in under reporting of breast cancers in cisgender males [39]. Although there are no population-based studies that document incidence of breast cancer in transgender patients, in a study from the Netherlands following a cohort of 2,307 transgender patients, breast cancer was diagnosed in one transgender male and in two transgender females. The authors calculated that these findings translated into a cancer rate of 4.1 per 100,000 life years in transgender females and 5.9 per 100,000 transgender males, similar to the approximately 1.2 in 100,000 cisgender males and significantly lower than the rate of 170 in 100,000 in cisgender females [40]. These authors concluded that the risk for breast cancer in male to female patients is similar to natal male sex and breast cancer risk in female to male patients also is quite low.

A descriptive study performed in the U.S. published in 2015 measured breast cancer incidence in a cohort of 5,135 transgender veterans [41]. Chart review revealed three cancers in transgender women and seven cancers in transgender men yielding a rate of 20 per 100,000 years. The three transgender women all had advanced disease which the authors use as evidence for the need for screening using standard guidelines. One of the seven transgender men had undergone mastectomy including chest contouring. These data may be unreliable because terminology was not uniform, there was a lack of follow-up outside the VA system, and no specific data on hormone use were provided.

### Transgender Women

Apart from the prospective and retrospective cohorts from the Netherlands and U.S. referred to above, there are a small number of case reports of breast cancers in transgender women. As of 2014, there were ten reported cases of breast cancer in transgender women on hormone treatment. Among those ten cases we observed that age at diagnosis tended to be younger than in cisgender populations, with a median age 48 compared to age 61 in cisgender females. In addition, five out of eight cases had ER negative cancer, and lobular development was similar to that of an adolescent girl [17]. Our series included a case of DCIS detected by screening, one of the few such screen detected cancers in the literature [17]. This highlights the need for more data to assess the role of screening in this population. It is likely that the low incidence of breast cancers is a result of under reporting.

### Transgender Men

It appears that risk for breast cancer in trans men on testosterone treatment is low and it has been hypothesized that testosterone treatment reduces risk [42•]. There are five case reports of breast cancer in transgender men all of whom were treated with testosterone. Of these five cases, four of the cancers were ER positive and three were PR negative. Invasive duct cancers that were ER + PR- were found in two trans men who had not had mastectomy, one at age 27 and the second at age 53 [43]. The three additional invasive duct cancers were residual breast tissue in trans men following subcutaneous mastectomy. One was in the nipple and the second was areolar diagnosed at age 33 following 13 years of hormone treatment and at age 42 following 1 ½ years on hormone treatment, respectively [44, 45]. The third case is a 41 year old who developed invasive duct carcinoma in the left lower outer quadrant after 15 years on hormone treatment [46].

## Breast Cancer Screening

### Transgender Men

It is generally recommended that transgender men who have not had mastectomy or who have only undergone breast reduction should follow the same guidelines for screening mammography as cisgender women irrespective of hormone treatment [47].

While not supported by data, transgender men who have had mastectomy with chest contouring should consider clinical chest exams because, as noted above, cancers have been reported in this setting. Mammography may not be feasible in this group.

### Transgender Women

There is a lack of consensus among the many organizations that provide recommendations for screening mammography for transgender women who are on hormone treatment. Screening mammography recommendations range from annual or biennial generally starting at age 50 for transgender women who have received estrogen treatment for at least 5 years [47, 48]. Other risk considerations include body mass index over 35 and a family history of breast cancer [47, 48]. Some organizations simply suggest screening all transgender women identically to cisgender women [7•]. There is no evidence base for these recommendations.

There are limited conflicting observations of utilization of mammography by transgender individuals. Using phone surveys conducted in 2014 by the Center for Disease Control’s Behavioral Risk Factor Surveillance System including 220 respondents, 54.5% of transgender females and 64.3% of transgender male respondents had undergone mammography within the past year [49]. This study lacked information about hormone and surgical treatment and involved a small number of participants. A small retrospective study which compared mammography utilization between cisgender women, transgender women on at least 5 years of hormone treatment, and preoperative transgender men at a single urban health center, observed decreased utilization of mammography by both transgender men and women [50].

### Additional Risk Considerations

While any patient with breast tissue may develop breast cancer, risks vary according to genotype, phenotype, and acquired risk factors due to certain exposures. Table 2 outlines risks and screening recommendations for various groups. It is important for transgender individuals to receive detailed genetic risk assessment based on family history of breast, ovarian, prostate, and/or pancreatic cancer and ancestry (e.g., Ashkenazi heritage), as these factors correlate with incidence of breast cancer susceptibility mutations. A major driver of risk for breast cancer is inherited mutations of genes such as BRCA2, BRCA1 and more rarely PALB2 and CHEK2 [51]. While cisgender men have a lifetime risk for breast cancer of 0.1%, those with Klinefelter’s syndrome or a deleterious mutation of the BRCA2 gene have an elevated lifetime risk for breast cancer of 5–7% [52, 53]. How hormone treatment might alter risk in the setting of a deleterious gene mutation remains to be determined. It has been suggested that transgender women with a BRCA gene mutation should use the same screening guidelines as cisgender mutation carriers [54].

**Table 2:** Breast cancer risk and screening recommendations

Group	Lifetime risk for breast cancer	Suggested ages to start mammography per various guidelines	Interval	Note
Cisgender women at average risk	12%	Age 40 [56] Age 50 [57]	Annual [56] Biennial [57]	Recommendations on age to start and interval varies across organizations
Cisgender men at average risk	0.1%	N/A	N/A	
Transgender women on at least 5 years of hormone treatment	Slightly higher than cisgender men	50 [48] 50 [49] Follow non-transgender guidelines [7, 59]	Biennial [48] Annual [49]	Consider additional risk factors: BMI > 35, family history of breast cancer [48, 49]
Transgender men without mastectomy (no “top surgery”)	12%	Same recommendation as cisgender women [47]	Annual	Risk may vary with hormone treatment or oophorectomy
Klinefelter’s syndrome [52]	3–7.5%	N/A	N/A	Consider annual clinical breast exam (CBE) at age 35 [60]
Transgender men post top surgery on hormone treatment	Slightly higher than cisgender male	N/A	N/A	Chest awareness. Annual chest wall and axillary exam [58]
Cisgender men w BRCA2 gene mutation [53]	5–7%	N/A	N/A	Breast awareness. Annual CBE at age 35 [58]
Cisgender men w BRCA1 gene mutation [53]	1.2%	N/A	N/A	
Cisgender women w BRCA 1 or 2 gene mutation [53]	26–91% (> 20%)	Age 25	Annual	Annual CBE at age 25 [58] Annual breast MRI starting at age 25 [58]

Because there is no evidence base for screening recommendations, any recommended screening should be done as part of an ongoing prospective study. We have initiated one such effort in New York, which is open to any individual seeking more information [55]. Additional prospective data pertaining to transgender populations incorporating phenotype, genotype, acquired risks, hormone exposure, and correlating radiographic features and clinical outcome is critically needed to develop risk-adapted breast cancer screening protocols in this population.

## Conclusion

Radiologists need to be knowledgeable about hormone and surgical treatments for transgender individuals that influence imaging appearance and risk for breast diseases. Imaging should be performed in a culturally sensitive environment. The current recommendations for mammography screening are based on data from cisgender women. Additional data, specifically from prospective longitudinal follow-up of transgender individuals electing breast cancer radiologic surveillance is needed to understand breast cancer risk in this population and to develop appropriate risk-adapted screening protocols. We have initiated one such effort in New York.
